# *Rhodotorula glutinis* Phenylalanine/Tyrosine Ammonia Lyase Enzyme Catalyzed Synthesis of the Methyl Ester of *para*-Hydroxycinnamic Acid and its Potential Antibacterial Activity

**DOI:** 10.3389/fmicb.2016.00281

**Published:** 2016-03-08

**Authors:** Marybeth C. MacDonald, Pugazhendhi Arivalagan, Douglas E. Barre, Judith A. MacInnis, Godwin B. D’Cunha

**Affiliations:** ^1^Department of Chemistry, Cape Breton UniversitySydney, NS, Canada; ^2^Department of Environmental Biotechnology, School of Environmental Sciences, Bharathidasan UniversityTiruchirappalli, India; ^3^Department of Health Sciences and Emergency Management, Cape Breton UniversitySydney, NS, Canada

**Keywords:** *Rhodotorula glutinis*, phenylalanine/tyrosine ammonia lyase, enzyme catalysis, methyl ester of *para*-hydroxycinnamic acid, antibacterial activity

## Abstract

Biotransformation of L-tyrosine methyl ester (L-TM) to the methyl ester of *para*- hydroxycinnamic acid (*p*-HCAM) using *Rhodotorula glutinis* yeast phenylalanine/tyrosine ammonia lyase (PTAL; EC 4.3.1.26) enzyme was successfully demonstrated for the first time; progress of the reaction was followed by spectrophotometric determination at 315 nm. The following conditions were optimized for maximal formation of *p*-HCAM: pH (8.5), temperature (37°C), speed of agitation (50 rpm), enzyme concentration (0.080 μM), and substrate concentration (0.50 mM). Under these conditions, the yield of the reaction was ∼15% in 1 h incubation period and ∼63% after an overnight (∼18 h) incubation period. The product (*p*-HCAM) of the reaction of PTAL with L-TM was confirmed using Nuclear Magnetic Resonance spectroscopy (NMR). Fourier Transform Infra-Red spectroscopy (FTIR) was carried out to rule out potential hydrolysis of *p*-HCAM during overnight incubation. Potential antibacterial activity of *p*-HCAM was tested against several strains of Gram-positive and Gram-negative bacteria. This study describes a synthetically useful transformation, and could have future clinical and industrial applications.

## Introduction

Enzymes of the ammonia-lyase family, such as histidine ammonia lyase (HAL) and PAL, catalyze the deamination of amino acids (Poppe and Rétey, 2005). PAL (EC 4.3.1.24) activity has been demonstrated in plants, fungi, yeasts, and a number of microbial species including *cyanobacteria*, *Photorhabdus luminescens*, *Sorangium cellulosum*, and *streptomyces* (Fritz et al., 1976; Hanson and Havir, 1979; Williams et al., 2005; Hyun et al., 2011; Babaoǧlu Aydaş et al., 2013; Cui et al., 2013, 2014). PAL is the first enzyme in the phenylpropanoid sequence, a secondary metabolic pathway, in plants; this pathway is involved in the synthesis of important compounds including coumarins (have antimicrobial properties), lignins (used for structural support) and flavonoids (colorful component of many flowers; Koukol and Conn, 1961). PAL has a catabolic role in fungi, allowing the microbe to use L-Phe/L-Tyr as the sole source of carbon and nitrogen; in bacteria, the enzyme is involved in the synthesis of secondary metabolites (Hyun et al., 2011; Cui et al., 2014). PAL has not been found to date in animal tissues, including humans.

Phenylalanine ammonia lyase from different species including *Rhodotorula glutinis* yeast shows activity toward a broad range of phenylalanine analogs (MacDonald and D’Cunha, 2007). PAL catalyzes the transformation of L-Phe to *t*-CA and ammonia (Koukol and Conn, 1961). PAL has also been shown to accept L-PM as the substrate (D’Cunha et al., 1994). In addition, PAL reverse reactions for the synthesis of L-Phe from *t*-CA and L-PM from *t*-CM have been successfully demonstrated (Yamada et al., 1981; Evans et al., 1987a; D’Cunha et al., 1994). The enzyme is shown to have specificity for L-Tyr, that is, PAL can produce *p*-HCA by deamination of tyrosine (Neish, 1961). PAL enzymes that demonstrate specificity for L-Tyr are referred to as tyrosine ammonia lyases (TAL; EC 4.3.1.25); and the enzymes that show dual specificity toward L-Phe and L-Tyr are called PTAL (EC 4.3.1.26; Neish, 1961; Xue et al., 2007a).

**FIGURE 1:**
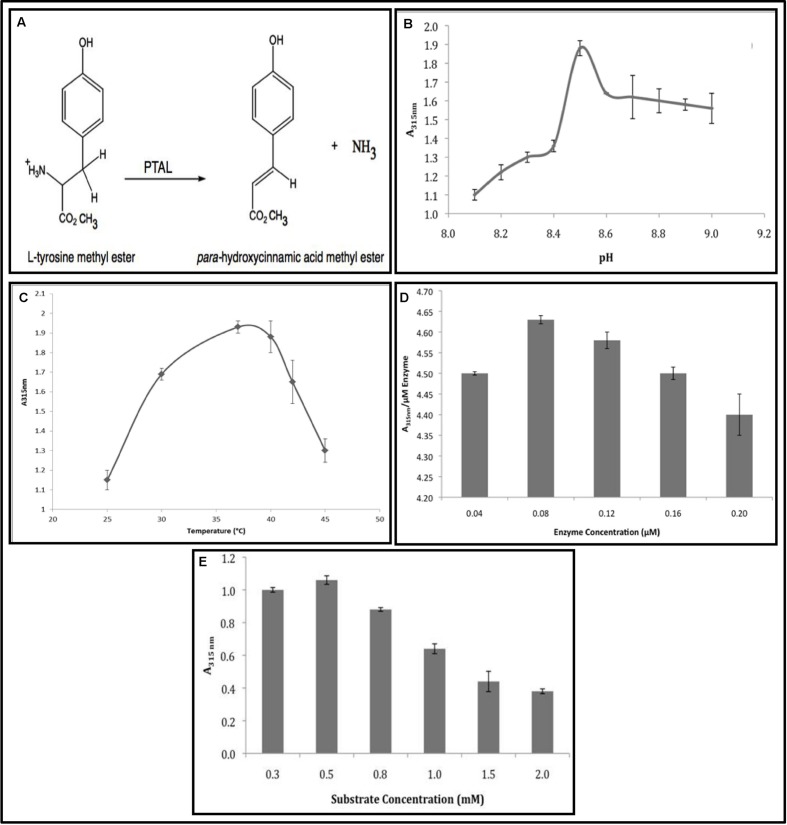
***Rhodotorula* PTAL catalyzed the biotransformation of L-TM to *p*-HCAM. (A)** The PTAL Reaction. **(B)** Effect of pH. **(C)** Effect of temperature. **(D)** Effect of enzyme concentration. **(E)** Effect of substrate concentration. PTAL activity is reported in terms of *p*-HCAM formation (measured as O.D. units at 315 nm).

The present study is an extension of the earlier work on PAL/PTAL catalyzed synthesis of important molecules and their applications (Neish, 1961; Yamada et al., 1981; Evans et al., 1987a; D’Cunha et al., 1994; Burchard et al., 2000; Solecka and Kacperska, 2003; Bouzid et al., 2006; Xue et al., 2007a,b; Dogbo et al., 2012) with the main objective being testing the efficacy of using *R. glutinis* PTAL in the biotransformation of L-TM to the methyl ester of *p*-HCAM according to the reaction shown in **Figure 1A**.

There are a number of reports on the synthesis cinnamic acid/ coumaric acid derivatives including the synthesis of *p*-HCAM (Venkateswarlu et al., 2006; Stankova et al., 2009; Khatkar et al., 2014). However, the procedures for *p*-HCAM formation employed lengthy and time-consuming multi-step methods with resultant low yields. There is therefore a need to develop a simple and rapid procedure for the production of *p*-HCAM, which has recently been shown to exhibit antimicrobial, antiviral and antioxidant activity (Proestos et al., 2006; Venkateswarlu et al., 2006; Stankova et al., 2009; Terpinc et al., 2011; Chochkova et al., 2013; McCarthy et al., 2013; Khatkar et al., 2014).

We have successfully demonstrated the direct one-step PTAL catalyzed synthesis of *p*-HCAM and confirmed that it shows antibacterial activity against several pathogenic bacteria. Derivatives of *p*-HCA, such as ferulic acid, have important roles in plants (Burchard et al., 2000), health and pharmaceutical applications (Burchard et al., 2000; Solecka and Kacperska, 2003), and food industry (Bouzid et al., 2006). Therefore, in addition to using *p*-HCAM as an antibacterial agent (perhaps as a topical treatment agent or disinfectant), we also intend testing its potential application as a food additive (inclusion in canned foods to prevent microbial contamination).

## Materials and Methods

### Enzyme Source

*Rhodotorula glutinis* yeast was used as the source of PTAL (E.C.4.3.1.26) enzyme. The isolated enzyme was obtained from Sigma–Aldrich Company (St. Louis, MO, USA).

### Chemicals

Authentic L-Phe, L-PM, L-Tyr, and L-TM were obtained from Sigma–Aldrich Canada Ltd (Oakville, ON, Canada). Authentic *p*-HCA and *p*-HCAM were procured from Fisher Scientific (Whitby, ON, Canada) and Frinton Laboratories (Hainesport, NJ, USA), respectively. Deuterated solvents used for NMR studies were purchased from Acros Organics (Morris Plains, NJ, USA). All other commonly used chemicals, reagents, and solvents were of highest analytical grade; and were bought from Fisher Scientific (Whitby, ON, Canada).

### PTAL Assay

The PTAL assay using L-TM as the substrate was performed and formation of the product, *p*-HCAM, was followed spectrophotometrically, using a Cary 100 UV-vis spectrometer, by monitoring the wavelength at 315 nm. Initially, the PTAL reaction was carried out as follows: the reaction mixture (1.0 mL) containing 50.0 mM Tris-HCl buffer (pH 8.8), 1.0 mM L-TM, and 0.1 μM PTAL enzyme protein was incubated at room temperature for 60 min. At the end of the incubation period, the reaction was terminated by deactivating the enzyme with 20 μL of 12 M HCl. The reaction mixtures, in which substrate and enzyme were added, after termination of reaction, served as substrate and enzyme controls, respectively. PTAL activity is reported in terms of *p*-HCAM formation (measured as O.D. units at 315 nm). Different conditions for maximal *p*-HCAM production including pH, temperature, speed of mixing the reaction mixture, and the enzyme and substrate concentrations were then standardized when the reaction was carried out for 60 min.

### Analysis of *para*-Hydroxycinnamic Acid Methyl Ester by NMR and FTIR

The PTAL catalyzed biotransformation of L-TM to *p*-HCAM was scaled up to a total volume of 5.0 mL (50.0 mM Tris-HCl buffer pH 8.5, 0.50 mM L-TM, and 0.080 μM PTAL enzyme protein), and the reaction was carried out for ∼18 h to ensure production of sufficient amount of the product for detection by NMR and FTIR. At the end of the incubation period, solvent buffer from the reaction mixture was removed by rotary evaporation.

The residue obtained at the end of the rotary evaporation step was treated with deuterated chloroform (*p*-HCAM was soluble, whereas L-TM and PTAL were insoluble in CDCl_3_), and the solution was filtered to separate unreacted substrate and enzyme from the product. The filtrate containing *p*-HCAM was then characterized using solid state CP/MAS NMR carried out on a Bruker Avance NMR spectrometer with a 9.4 T magnet (400.24 MHz proton Larmor frequency and 100.65 MHz ^13^C Larmor frequency). Authentic *p*-HCAM in CDCl_3_ was characterized in a similar manner by NMR.

Fourier Transform Infra-Red spectroscopy analysis was carried out to rule out potential hydrolysis of *p*-HCAM during overnight incubation; we analyzed authentic *p*-HCA and *p*-HCAM, and the product of our reaction (*p*-HCAM in CDCl_3_ was retrieved by rotary evaporation of the solvent). KBr pellets were prepared using these specimens; and infrared absorption spectra of each were measured by absorption techniques using a Nicolet Impact 410 FTIR over a range of 4000–490 cm^-1^.

### Potential Antibacterial Activity of *Para*-Hydroxycinnamic Acid Methyl Ester

Biotransformation of L-TM to *p*-HCAM using PTAL was carried out according to the protocol developed in our lab; and the residue obtained was dissolved in 50% ethanol. Unreacted L-TM and PTAL which were insoluble in ethanol were separated out by filtration, and the antibacterial activity of the filtrate containing *p*-HCAM was tested by cylinder-plate (agar-cup plate) diffusion method (Cooper and Kavanaugh, 1972) against Gram-positive organisms (*Bacillus cereus* and *Staphylococcus aureus*) and Gram-negative organisms (*Escherichia coli*, *Klebsiella pneumoniae*, *Proteus vulgaris* and *Pseudomonas aeruginosa*). Each of the bacterial cultures was grown for 24 h at 37°C on 2.8% nutrient agar plates. Suspensions of each culture were prepared in sterile saline and then swabbed onto 3.8% Muller-Hinton agar plates. Various concentrations of *p*-HCAM solution (1.0, 3.0, and 5.0%) were tested for antibacterial activity against each culture. The Muller-Hinton agar plates were incubated for 24 h at 37°C and the zones of inhibition were measured.

### Data Analysis

The values reported are a mean of at least five independent determinations.

## Results

In this study, we have developed a simple and rapid procedure for the production of *p*-HCAM and shown its potential antibacterial activity.

### Solubility and Maximum Wavelength of L-Tyrosine Methyl Ester and *para*-Hydroxycinnamic Acid Methyl Ester

The solubility of L-TM and *p*-HCAM was tested in several buffer systems, reagents, and solvents (data not shown). Due to limited solubility of L-TM at low pH, PTAL activity was determined between 8.1 and 9.0 using 1 mM substrate concentration. Since L-TM and *p*-HCAM have distinct λ_max_ at 275 and 315 nm, respectively; it can be concluded that interference of substrate with product determination is unlikely to happen. Further confirmation that substrate and enzyme do not contribute to absorbance values when determining the product was carried out by using appropriate substrate and enzyme controls during PTAL assay. Using an enzyme control was particularly important as the PAL/PTAL enzyme shows absorbance at 280 nm.

### Optimal Conditions for PTAL Catalyzed Biotransformation of L-Tyrosine Methyl Ester to *para*-Hydroxycinnamic Acid Methyl Ester

Several experimental conditions including pH, temperature, speed of mixing the reaction milieu, and enzyme and substrate concentrations were optimized when this reaction was carried out for an incubation period of 60 min.

### Effect of pH

One of the most important factors influencing enzyme activity is pH. Since, L-TM was totally insoluble in 0.1 M phosphate buffers at pH 7.0 and 8.0 but soluble in 0.1 M Tris-HCl buffer at pH 9.0, we carried out pH optimization studies using Tris-HCl buffer with increments of 0.1 units in the range 8.1–9.0. PTAL catalyzed transformation of L-TM to *p*-HCAM was maximal at pH 8.5 (**Figure 1B**). We did not test pH exceeding 9.0; PAL has been reported to show a progressive gradual decline in activity at pH greater than 9.0 (Yamada et al., 1981; Evans et al., 1987a; D’Cunha et al., 1994). It is pertinent to mention here that L-TM is totally insoluble in Tris-HCl buffer at concentrations of 0.020 M or more.

### Effect of Temperature

From the curve of temperature versus enzyme activity (**Figure 1C**), it is evident that in the present study, optimal PTAL activity was obtained at 37°C and that there was progressive decline in enzyme activity at higher temperatures.

### Effect of Speed of Agitation

There was no pronounced effect on PTAL enzyme activity when the speed of agitating the reaction mixture during the incubation period was increased above 50 rpm (**Table 1**). These results correspond well with almost all studies on effect of agitation on PAL protein using L-Phe, L-PM, *t*-CA, and *t*-CM substrates; optimal enzymatic conversions have been obtained at ∼50–100 rpm (Yamada et al., 1981; Evans et al., 1987a,b; D’Cunha et al., 1994, 1996; Quinn et al., 2011).

**Table 1:** Effect of speed of agitation on the synthesis of *p*-HCAM by *Rhodotorula* PTAL.

Speed of agitation (rpm)	*A*_315nm_ 1.51	Standard deviation
0		±0.04
25	1.63	±0.02
50	1.98	±0.06
100	1.97	±0.07
150	1.89	±0.07
200	1.82	±0.15

*Phenylalanine/tyrosine ammonia lyase activity is reported in terms of p-HCAM formation (measured as O.D. units at 315 nm).*

### Effect of Enzyme and Substrate Concentration

Unit Enzyme activity (*A*_315__nm_/1.0 μM TAL) was maximal when 0.080 μM PTAL enzyme protein was used for assay (**Figure 1D**). There was no substantial increase in PTAL enzyme activity beyond this concentration of enzyme protein. The enzyme activity for individual concentrations of L-TM is shown in **Figure 1E**. Unit Enzyme activity (*A*_315__nm_/1.0 mM L-TM) was maximal when 0.50 mM L-TM was used for assay; PTAL enzyme activity decreased progressively below and above this substrate concentration.

### Percent Yield of *para*-Hydroxycinnamic Acid Methyl Ester under Optimal Conditions

The conditions standardized for the optimal production of *p*-HCAM by PTAL are summarized in **Table 2**. Under these optimized conditions, ∼15% transformation of L-TM to *p*-HCAM was obtained in 60 min. The amount of *p*-HCAM (μmoles) was obtained from *A*_315__nm_ values by using a calibrated curve constructed with authentic *p*-HCAM drawn from a 0.010 M stock solution (*y* = 0.00034929x^2^ + 0.00013286x + 0.02900000, *R*^2^ = 0.99893022). The yield of *p*-HCAM increased to ∼63%, when the reaction was carried out overnight (∼18 h).

**Table 2:** Optimized reaction conditions for PTAL catalyzed transformation of L-TM to *p*-HCAM.

Parameter	Range tested	Optimal value
pH	8.1–9.0	8.5
Temperature	25–45°C	37°C
Speed of agitation	0–200 rpm	50 rpm
Enzyme concentration	0.04–0.2 μm	0.08 μm
Substrate concentration	0.25–2.0 mM	0.50 mM

*Phenylalanine/tyrosine ammonia lyase activity is reported in terms of p-HCAM formation (measured as O.D. units at 315 nm).*

### Nuclear Magnetic Resonance Spectroscopy (NMR)

**FIGURE 2:**
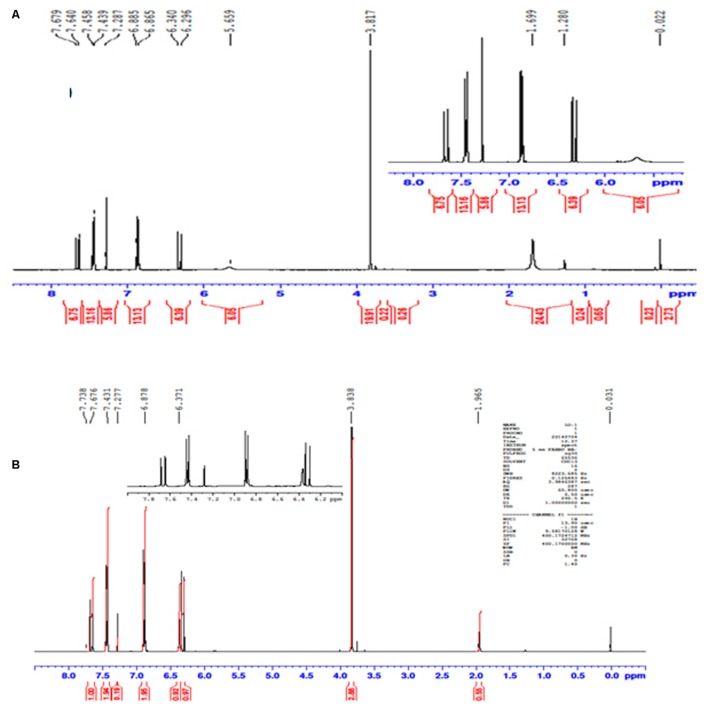
**Profile of ^1^H NMR spectra. (A)** Product of PTAL catalyzed biotransformation of L-TM. **(B)** Authentic *p*-HCAM. *p*-HCAM was characterized using solid state CP/MAS NMR carried out on a Bruker Avance NMR spectrometer with a 9.4 T magnet (400.24 MHz proton Larmor frequency).

**FIGURE 3:**
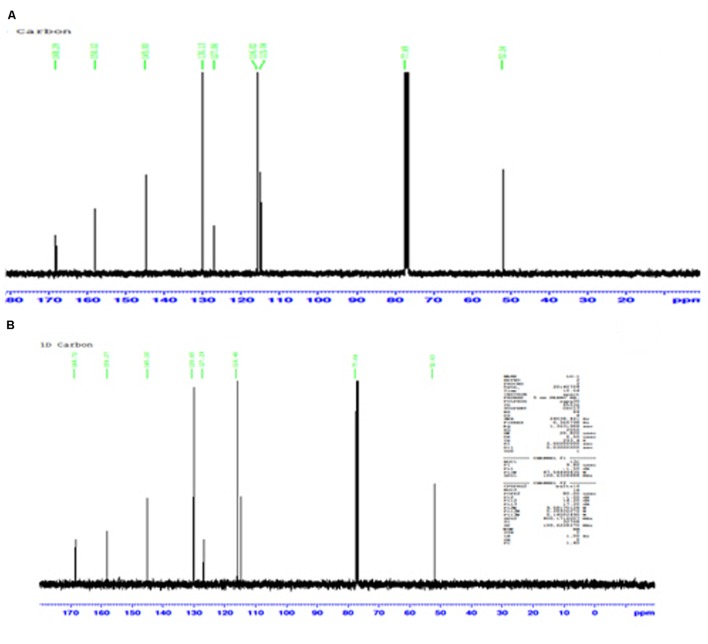
**Profile of ^13^C NMR spectra. (A)** Product of PTAL catalyzed biotransformation of L-TM. **(B)** Authentic *p*-HCAM. *p*-HCAM was characterized using solid state CP/MAS NMR carried out on a Bruker Avance NMR spectrometer with a 9.4 T magnet (100.65 MHz ^13^C Larmor frequency).

**SCHEME 1:**
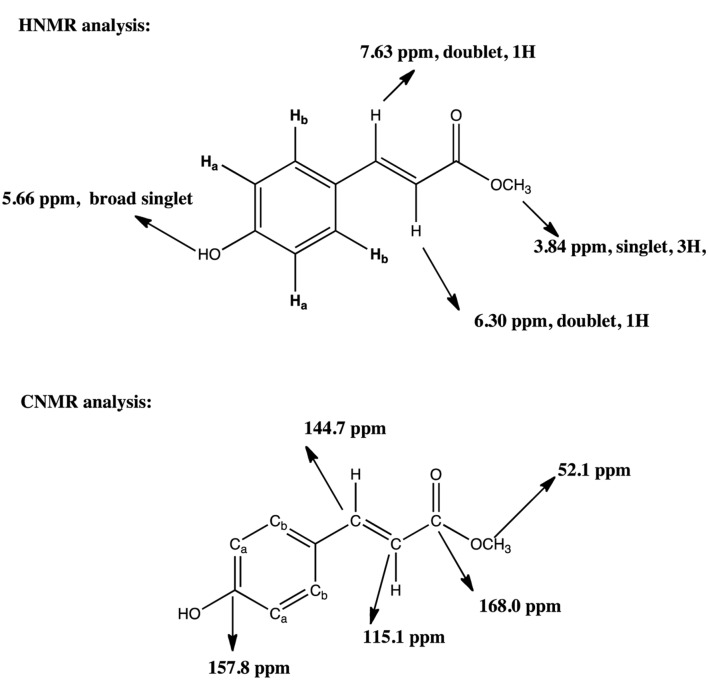
**^1^H NMR and ^13^C NMR analysis of synthesis of p-HCAM by Rhodotorula PTAL.** HNMR analysis: The aromatic protons Ha will be at 7.44 ppm, 2H, doublet and Hb will be at 6.3 ppm, 2H, doublet As usual, the peak at 7.29 ppm is the solvent peak for CDC1_3_. CNMR analysis: The carbons Ca will be same and are at 1155 ppm and Cb are both at 130.1 ppm. From molecular formula, 10 carbons but due to symmetry, only 8 carbons observed. As usual, the peak at 77.6 ppm is the solvent peak for CDC13

The conversion of L-TM to *p*-HCAM reported in this study is a novel reaction. NMR acquired data provides convincing evidence for the formation of the desired product, *p*-HCAM. The ^1^H (**Figures 2A,B**) and ^13^C (**Figures 3A,B**) NMR spectra of the product of our reaction and of authentic *p*-HCAM were identical. The analysis of NMR spectral peaks representing different protons and carbons of the product is shown in **Scheme 1**.

### Fourier Transform Infra-Red Spectroscopy (FTIR)

**FIGURE 4:**
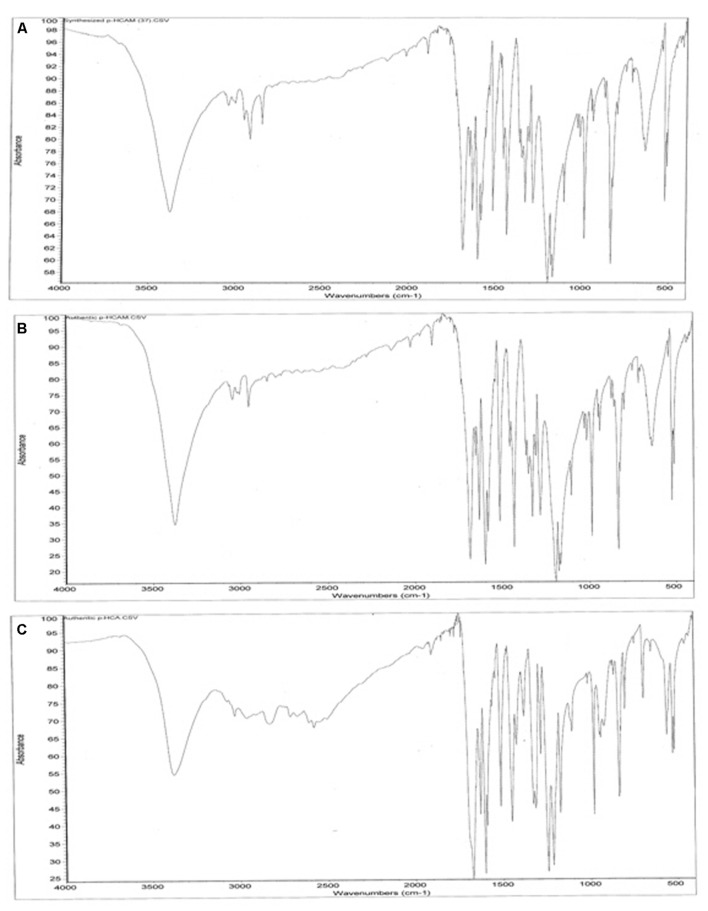
**Profile of FTIR spectra. (A)** Product of PTAL catalyzed biotransformation of L-TM. **(B)** Authentic *p*-HCAM. **(C)** Authentic *p*-HCA. Infrared absorption spectra of KBr pellets of these specimens were obtained using a Nicolet Impact 410 FTIR over a range of 4000–490 cm^-1^.

From the striking similarities between FTIR spectra of the product of our reaction and authentic *p*-HCAM (**Figures 4A–C**), it is further confirmed that the PTAL catalyzed biotransformation of L-TM to *p*-HCAM was successful. The FTIR spectral profiles reveal that product obtained in our study and authentic *p*-HCAM have nearly identical absorbance in the range 3700–2000cm^-1^; while authentic *p*-HCA gives a significantly different absorbance profile in this region. Therefore, we can conclude that there is negligible or no hydrolysis of *p*-HCAM to *p*-HCA during the incubation period of ∼18 h.

### Antibacterial Activity of *para*-Hydroxycinnamic Acid Methyl Ester

The zones of inhibition obtained with *P. vulgaris* and *B. cereus* (**Table 3**) when 5.0% *p*-HCAM (product of PTAL obtained in our lab) was loaded in the well of Muller-Hinton agar plate, were comparable with that obtained with standard antibiotic, Ampicillin (∼9 mm zone of inhibition). While substantial antibacterial activity was obtained against *S. aureus*, *E. coli*, and *K. pneumoniae*, the antibacterial effect against *P. aeruginosa* was negligible (we have not tried a concentration exceeding 5.0% *p*-HCAM in this study). Ethanol in which the product of our reaction was dissolved, did not contribute to the antibacterial activity (no zone of inhibition was obtained). The data obtained with 1.0, 3.0, and 5.0% concentrations of authentic *p*-HCAM (commercially obtained from Frinton Laboratories, Hainesport, NJ, USA) revealed similar results with each of the six strains used in this study; however, smaller zones of inhibition (data not shown in **Table 3**) were obtained, possibly due to the presence of some impurities.

**Table 3:** Antibacterial activity of *p*-HCAM obtained using PTAL (zone of inhibition in mm).

Bacterial cultures	1.0% *p*-HCAM	3.0% *p*-HCAM	5.0% *p*-HCAM
*B. cereus*	1 mm	5 mm	7 mm
*S. aureus*	–	1 mm	5 mm
*E. coli*	–	1 mm	6 mm
*K. pneumoniae*	–	1 mm	5 mm
*P. vulgaris*	4 mm	7 mm	8 mm
*P. aeruginosa*	–	–	–

*–indicates, no significant antibacterial activity. The solvent in which p-HCAM was dissolved, ethanol, did not show antibacterial activity (no zone of inhibition was obtained). Standard antibiotic, Ampicillin, gave ∼9 mm zone of inhibition.*

## Discussion

Enzymatic synthesis of industrially relevant biomolecules is an exciting and emerging area in modern biotechnology. PAL/PTAL has many potential applications including production of L-Phe, L-PM, and *p*-HCA (Neish, 1961; Yamada et al., 1981; Evans et al., 1987a; D’Cunha et al., 1994; MacDonald and D’Cunha, 2007; Xue et al., 2007a). There is great demand for the synthesis of pure L-Phe, L-PM, and *p*-HCA: L-Phe is an essential amino acid used in preparing dietary protein supplements; L-PM is one of the two precursors of the non-calorific sweetener aspartame (L-aspartyl-L-PM); and *p*-HCA has several applications in pharmaceutical, health, and food industries (Burchard et al., 2000; Solecka and Kacperska, 2003; Bouzid et al., 2006; MacDonald and D’Cunha, 2007; Xue et al., 2007a,b; Dogbo et al., 2012). We have successfully demonstrated PTAL catalyzed conversion of L-TM to *p*-HCAM for the first time; the one-step enzymatic procedure reported here would be a synthetically useful transformation for potential applications in pharmaceutical, health, and food industries.

Almost all previous studies on effect of pH on PAL/PTAL activity have reported that the optimum pH is in the range of 8.5–9.0; and our study (**Figure 1B**) was no exception. The only discrepancy was that we did not get a typical bell shaped curve for a plot of pH vs enzyme activity as reported in earlier studies (Neish, 1961; Yamada et al., 1981; Evans et al., 1987a,b; D’Cunha et al., 1994; Xue et al., 2007a). This discrepancy could result from unique interaction between substrate and enzyme at pH 8.5 during the formation of *p*-HCAM. In the present study; it was found that there is sharp increase in enzyme activity when the pH is raised from 8.4 to 8.5; and there is a gradual decline when the pH is 8.6 or greater. One conclusion we can draw from this result is that protonation and deprotonation of enzyme protein over a narrow pH range has profound influence on formation of *p*-HCAM from L-TM by PTAL.

The results of correlation of temperature with PTAL activity for the synthesis of *p*-HCAM obtained in this study (**Figure 1C**) are similar to most results obtained with temperature studies on PAL/PTAL (including *R. glutinis* PAL/PTAL) that have been reported earlier, in that, the enzyme has been known to have maximal activity at 30–37°C, and that activity rapidly falls at temperatures equal to or greater than 40°C (Yamada et al., 1981; Evans et al., 1987b; D’Cunha et al., 1994; Ritenour and Saltveit, 1996; Solecka and Kacperska, 2003; Dogbo et al., 2012). In addition, it has also been shown that PAL/PTAL protein is stable over long incubation periods (∼16–24 h) at temperatures ranging from 30 to 37°C (Evans et al., 1987b; D’Cunha et al., 1994; Ritenour and Saltveit, 1996; Dogbo et al., 2012). Therefore we can conclude that in the work reported here, PTAL stability is not compromised over ∼18 h incubation for the biotransformation of L-TM to *p*-HCAM.

One of the reasons for the progressive decrease in enzyme activity when the concentration of L-TM was increased to over 0.50 mM (**Figure 1E**) could be substrate inhibition of the enzyme. Several enzymes have been reported to be inhibited by their substrates when the latter are in high concentration; such type of inhibition is regarded as a form of uncompetitive allosteric inhibition of the enzyme (Garrett and Grisham, 2012). It is possible that something similar is taking place in this case.

The methods reported for the synthesis of *p*-HCAM are lengthy and tedious procedures involving combination of chemical and enzymatic steps. The methods reported by Venkateswarlu et al. (2006), Stankova et al. (2009), and Khatkar et al. (2014) have resulted in low yields of coumaric acid and cinnamic acid, and their derivatives. In contrast, a simple one-step synthesis of *p*-HCAM could be commercially attractive, in that, it would be a rapid and cost-effective procedure. Although PAL/PTAL have been extensively used in the synthesis of a number of compounds including optically pure molecules (Neish, 1961; Camm and Towers, 1973; Yamada et al., 1981; Evans et al., 1987a; Cheng and Breen, 1991; D’Cunha et al., 1994; Xue et al., 2007a) to the best of our knowledge, PTAL catalyzed synthesis of *p*-HCAM has not been shown before.

The results (**Table 3**) obtained for *S. aureus*, *E. coli*, and *P. aeruginosa* are in good agreement with those obtained by Venkateswarlu et al. (2006). However, it is not clear from their studies if the solvent in which *p*-HCAM was dissolved, contributed to the antibacterial activity. Since, *p*-HCAM is literally insoluble in aqueous media but freely soluble in organic solvents, and it is well known that a number of organic solvents exhibit antibacterial activity; it was ascertained that ethanol in which the product of our reaction was dissolved, did not contribute to the antibacterial activity (no zone of inhibition was obtained).

## Conclusion

The PTAL catalyzed biotransformation of L-TM to *p*-HCAM has been successfully demonstrated for the first time. Conditions were optimized for maximal *p*-HCAM production (the yield was calculated using a standard curve constructed using a known concentration of authentic *p*-HCAM); and the formation of *p*-HCAM was confirmed using NMR and FTIR. Among the six bacterial strains used in this study, *p*-HCAM exhibited remarkable antibacterial activity against *P. vulgaris* and *B. cereus*.

Further work in this direction would involve a systematic study on antimicrobial activity of *p*-HCAM against species known to cause a number of diseases in humans including pneumonia, meningitis, bronchitis, food poisoning, and botulism. We also intend exploring the potential of using *p*-HCAM as a topical treatment agent or disinfectant and food additive in canned food material. This data would be important from fundamental and applied perspectives.

## Author Contributions

The laboratory work was primarily carried out by MM and Dr. PA. The manuscript was written by Dr. PA and Dr. GD. GD was the mentor. Dr. DB and Ms. JM provided their expertise for experimental work and analysis of results.

## Conflict of Interest Statement

The authors declare that the research was conducted in the absence of any commercial or financial relationships that could be construed as a potential conflict of interest.

## Abbreviations

FTIR: Fourier Transform Infra-Red spectroscopy
L-Phe: L-phenylalanine
L-PM: L-phenylalanine methyl ester
L-TM: L-tyrosine methyl ester
L-Tyr: L-tyrosine
NMR: Nuclear Magnetic Resonance spectroscopy
PAL: Phenylalanine ammonia lyase
PTAL: Phenylalanine/tyrosine ammonia lyase
*p*-HCA: *para*-hydroxycinnamic acid
*p*-HCAM: methyl ester of *para*-hydroxycinnamic acid
*t*-CA: *trans* cinnamic acid
*t*-CM: *trans* cinnamic acid methyl ester
UV-Visible: Ultraviolet visible spectroscopy
